# Identification and characterization of human polyserase-3, a novel protein with tandem serine-protease domains in the same polypeptide chain

**DOI:** 10.1186/1471-2091-7-9

**Published:** 2006-03-27

**Authors:** Santiago Cal, Juan R Peinado, María Llamazares, Víctor Quesada, Angela Moncada-Pazos, Cecilia Garabaya, Carlos López-Otín

**Affiliations:** 1 Departamento de Bioquímica y Biología Molecular, Facultad de Medicina, Instituto Universitario de Oncología, Universidad de Oviedo, 33006-Oviedo, Spain

## Abstract

**Background:**

We have previously described the identification and characterization of polyserase-1 and polyserase-2, two human serine proteases containing three different catalytic domains within the same polypeptide chain. Polyserase-1 shows a complex organization and it is synthesized as a membrane-bound protein which can generate three independent serine protease domains as a consequence of post-translational processing events. The two first domains are enzymatically active. By contrast, polyserase-2 is an extracellular glycosylated protein whose three protease domains remain embedded in the same chain, and only the first domain possesses catalytic activity.

**Results:**

Following our interest in the study of the human degradome, we have cloned a human liver cDNA encoding polyserase-3, a new protease with tandem serine protease domains in the same polypeptide chain. Comparative analysis of polyserase-3 with the two human polyserases described to date, revealed that this novel polyprotein is more closely related to polyserase-2 than to polyserase-1. Thus, polyserase-3 is a secreted protein such as polyserase-2, but lacks additional domains like the type II transmembrane motif and the low-density lipoprotein receptor module present in the membrane-anchored polyserase-1. Moreover, analysis of post-translational mechanisms operating in polyserase-3 maturation showed that its two protease domains remain as integral parts of the same polypeptide chain. This situation is similar to that observed in polyserase-2, but distinct from polyserase-1 whose protease domains are proteolytically released from the original chain to generate independent units. Immunolocalization studies indicated that polyserase-3 is secreted as a non-glycosylated protein, thus being also distinct from polyserase-2, which is a heavily glycosylated protein. Enzymatic assays indicated that recombinant polyserase-3 degrades the α-chain of fibrinogen as well as pro-urokinase-type plasminogen activator (pro-uPA). Northern blot analysis showed that polyserase-3 exhibits a unique expression pattern among human polyserases, being predominantly detected in testis, liver, heart and ovary, as well as in several tumor cell lines.

**Conclusion:**

These findings contribute to define the growing group of human polyserine proteases composed at present by three different proteins. All of them share a complex structural design with several catalytic units in a single polypeptide but also show specific features in terms of enzymatic properties, expression patterns and post-translational maturation mechanisms.

## Background

The presence of several catalytic domains embedded in the same polypeptide chain is an unusual molecular feature, whose evolutionary or functional advantages are still unclear [1]. However, recent works have provided evidence that a number of proteins from different organisms exhibit these complex architectures. Among these multidomain enzymes there are luciferases, xylanases, chitinases, endoglucanases, kinases, and diverse hydrolases including proteases [2-9]. The occurrence of human proteases with different catalytic domains in the same translation product was first reported for two metalloproteinases: angiotensin-converting enzyme (ACE) and carboxypeptidase D [10,11]. ACE is a type-I membrane-bound metalloproteinase with two enzymatically active domains which shows specific catalytic constants and interact differently with several competitive inhibitors [12]. Carboxypeptidase D is also a type-I membrane metalloproteinase that contains three catalytic domains, two of them being catalytically active and showing optimal activities at different pHs [13]. More recently, and as part of our studies on mammalian degradomes – the entire protease complement of these organisms [14-17] –, we have identified and characterized a cDNA encoding an unusual mosaic protease called polyserase-1 [9] (polyserine protease-1). This protein shows a complex domain organization including a type-II transmembrane motif, a low-density lipoprotein receptor A module, and three tandem serine protease domains. Interestingly, analysis of post-translational processing mechanisms of polyserase-1 revealed that it is synthesized as a membrane-bound protein which undergoes a series of proteolytic processing events to generate three independent serine protease domains [9]. Further studies of the human degradome have revealed the occurrence of another gene coding for a protein with three tandem serine protease domains in a single polypeptide chain [3]. This protein – called polyserase-2 – is an extracellular glycosylated enzyme, whose three serine protease domains are not proteolytically cleaved and remain as an integral part of the same polypeptide chain. Enzymatic analysis of polyserase-2 has demonstrated that only its first protease domain is catalytically active, whereas the second and third domains are inactive due to the lack of critical residues present in the catalytic triad of serine proteases [3].

The finding of two polyserine proteases in the human genome, together with the presence of similar mosaic structures in serine proteases from other organisms such as *Xenopus laevis*, *Bufo japonicus*, *Drosophila melanogaster *and *Caenorhabditis elegans *[18-21], prompted us to explore the possibility that additional yet uncharacterized polyserases could be produced by human tissues. In this work, we report the finding of a novel human protein, tentatively called polyserase-3, which contains two serine protease domains in its amino acid sequence. We describe the molecular cloning of a full-length cDNA for this protein, and perform a structural and enzymatic analysis of this new protease. We also examine the expression pattern of polyserase-3 in human tissues and tumor cell lines, and perform a comparative analysis between this enzyme and the previously described polyserases-1 and -2 in terms of structural design, phylogenetic relationships, cellular location and post-translational maturation mechanisms. On the basis of the obtained results, we conclude that polyserase-3 is more closely related to polyserase-2 than to polyserase-1, but also exhibits a series of characteristic features unique for this novel polyprotein among all other polyserine proteases described to date.

## Results

### Identification and molecular cloning of a new human polyserine protease

**Figure 1:**
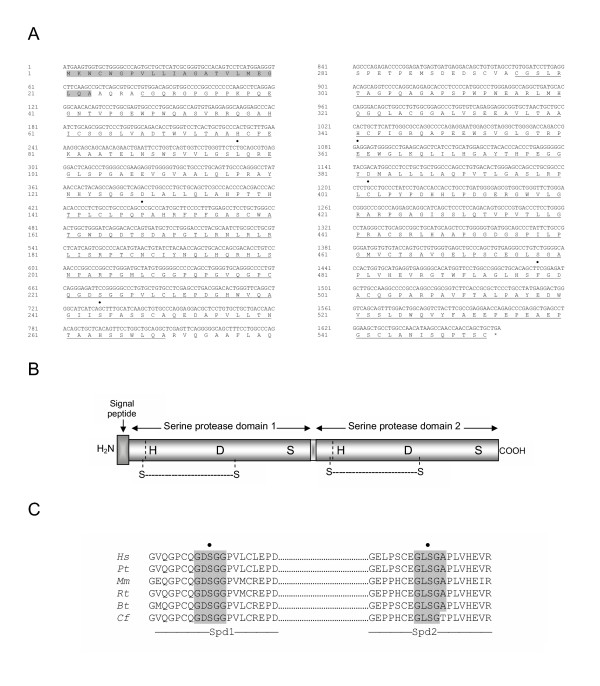
Deduced amino acid sequence and domain organization of human polyserase-3 cDNA. *A*, the deduced amino acid sequence is shown in single-code letter. The signal peptide is shaded in gray. The two serine-protease domains are underlined. The residues His, Asp and Ser, corresponding to the catalytic triad of the serine protease domains, are indicated with a black dot. *B*, schematic representation of the domain organization of polyserase-3. The two predicted disulfide bonds around the putative activation site of both serine protease domains are indicated. *C*, Amino acid sequence alignment around the catalytic serine residues of human polyserase-3 serine protease domains with equivalent sequences deduced from other species. *Spd*, serine protease domain.*Hs, Homo sapiens; Pt, Pan troglodytes, Mm, Mus musculus; Rn, Rattus norvegicus; Bt, Bos taurus; Cf, Canis familiaris*.

We used the polyserase-2 cDNA as a query and the BLAST algorithm to search regions in the human genome that could encode new proteases with several serine protease domains within the same polypeptide chain. This search allowed us to identify a region in chromosome 16p11.2 containing two serine protease domains closely linked. Conceptual translation of these domains showed that they were different to the serine protease domains of polyserase-2 and prostasin, which are serine protease genes located at the same chromosomal region [3,22]. Then, a PCR-based approach was designed to clone the cDNAs for these two uncharacterized serine protease domains. To this end, we used RNA from human liver and once the cloning process was completed, we confirmed that both domains were encoded by a single gene. Computer analysis of the obtained sequence revealed that this cDNA encodes a protein of 553 amino acids, with a predicted molecular mass of 58.4 kDa (Fig. 1A and EMBL accession number **AJ627035**). Following the nomenclature system proposed for these enzymes [9], we have tentatively called this new polyserine protease *polyserase-3*.

A detailed analysis of polyserase-3 sequence showed that it contains the structural hallmarks characteristic of serine proteases (Fig. 1A), with some relevant particularities. Thus, the sequence contains a signal peptide (positions 1 to 23), which predicts that this protein is targeted to the endoplasmic reticulum to direct its secretion outside of the cell. Following this region, the first serine protease domain (Spd1) can be recognized (positions 28 to 270) although this domain does not show the Arg-↓-Ile-Val-Gly-Gly consensus activation motif present in most of these enzymes. The sequence present instead at this region is Pro-Lys-Pro-Gln-Glu. The catalytic triad of Spd1 comprises the residues His77, Asp128, and Ser224. Following a short spacer region (positions 271 to 294), the second serine protease domain (Spd2) can be clearly identified (positions 295 to 553). This domain also lacks a consensus activation motif and its catalytic triad comprises the residues His341, Asp382, and Ser478. This last Ser residue is located within the sequence Gly-Leu-Ser-Gly-Ala (positions 476 to 480), which does not exactly match the consensus motif Gly-Asp-Ser-Gly-Gly found in this class of enzymes. There are also a number of cysteine residues in both protease domains of polyserase-3 which are conserved in serine proteases, including those located at positions 28 and 144 in Spd1, and 296 and 402 in Spd2. These residues could form two disulfide bonds which would determine that both protease domains remain linked to the polypeptide chain if a cleavage would take place at the activation site (Fig 1B). All these structural features can also be found in the amino acid sequence of putative orthologs of polyserase-3 predicted from the genome analysis of *Pan troglodytes *(99% identity), *Bos taurus *(80%), *Canis familiaris *(84%), *Mus musculus *(81%), and *Rattus norvegicus *(80%) (Fig. 1C).

### Comparative analysis of polyserase-3 with other serine proteases

**Figure 2:**
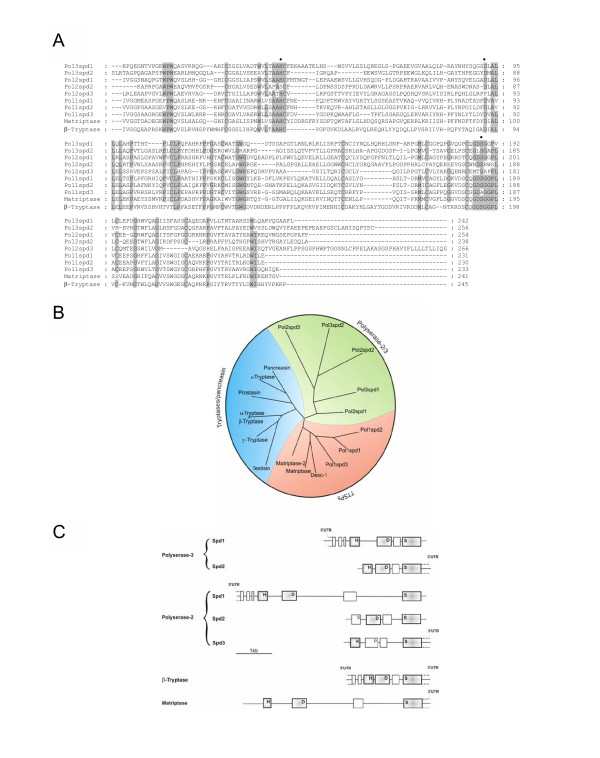
Relationship between polyserase-3 and other serine proteases. *A*, sequence comparison of the serine protease domains of polyserase-3 with other related proteins. Active site amino acids are marked with a black dot. Pol3Spd, pol2Spd and pol1Spd indicate the different serine protease domains of each polyserase. *B*, phylogenetic tree of different human serine proteases related to polyserase-3. The analysis was performed using the serine protease domain of each enzyme and the Phylogenic Interface Environment program supplied by the Human Genome Mapping Project. *C*, organization of the human polyserase-3 gene and comparison with other serine protease genes. Relative positions of each exon are indicated by boxes. In the case of matriptase, only the exon/intron organization of its serine protease domain is represented. *H*, *D *and *S *refer to positions of the codons that encode the catalytic triad amino acids in each protease gene.

The predicted amino acid sequence corresponding to the catalytic region of each polyserase-3 protease domain revealed a high degree of identity with other serine proteases (Fig 2). Comparative analysis of the first serine protease domain sequence indicated that the highest degree of identity was found with the first serine protease domain of polyserase-2 (40%). Significant percentage of identities were also found with pancreasin (36%), the second serine protease domain of polyserase-2 (35%), matriptase-2 (35%), prostasin (34%), and the third serine protease domain of polyserase-2 (34%). The second domain of polyserase-3 was also found to be closely related to the first protease domain of polyserase-2 (38%) as well as to other serine proteases such as γ-tryptase (37%), the second serine protease domain of polyserase-2 (34%), prostasin (33%), matriptase-2 (34%), and the first serine protease domain of polyserase-1 (32%). All these enzymes, with the exception of polyserase-2, belong to the transmembrane type (TTSP) or to the tryptase/pancreasin families of serine proteases [23-26]. Sequence alignments of these proteins with each protease domain of polyserase-3 (Fig. 2A) confirmed the extensive degree of conservation around the residues that form the catalytic triad of all these proteases. We also performed an analysis in the polyserase-3 sequence of molecular markers of serine protease evolution described by Krem and Di Cera [20]. This analysis revealed that polyserase-3 as well as polyserases- 1 and -2 use exclusively TCN codons for their active site serine residues (corresponding to Ser-195 in the chymotrypsinogen sequence). We also found that the Ser residues of all polyserases, which are equivalent to the Ser-214 residue of chymotrypsinogen, are always encoded by AGC codons. Finally, analysis of the third molecular marker associated with catalytic function in serine proteases (Pro or Tyr residues at position 225 in chymotrypsinogen numbering) revealed the presence of a Pro residue in both domains of polyserase-3. Likewise, Pro residues are present at the equivalent positions in the three serine protease domains of polyserase-1 as well as in the catalytically active domain of polyserase-2. Taken together, these results reinforce the classification of these polyproteases in the clan SA of serine proteases and extend the proposal of a close evolutionary relationship between them.

The phylogenetic tree for these proteins (Fig. 2B) also showed the close relationship of each polyserase-3 serine protease domain with the equivalent regions of polyserase-2. Together, the five protease domains of these two polyserases form a phylogenetic branch distantly related to the TTSP and tryptase/pancreasin families of serine proteases. Furthermore, the exon-intron organization of the catalytic region of the first domain of polyserase-3 is similar to that of TTSP and tryptase/pancreasin serine protease genes. In fact, the length of the intron that separates the exons containing the His and Asp residues of the catalytic triad of the first protease domain of polyserase-3, is similar to that found in the equivalent region of matriptase-2 [27,28]. However, the length of the remaining introns is similar to that found in the equivalent regions of the α/β-tryptases (Fig 2C). We have previously described that the polyserase-2 gene also shows a pattern of exon-intron organization that shares similarities with both groups of serine proteases [3]. Likewise, the polyserase-3 gene also contains three coding exons in the genomic region that comprises the signal sequence and the putative activation site. By contrast, only two coding exons are found in the equivalent region of α/β-tryptase genes [29-32].

### Molecular modeling of polyserase-3 serine protease domains

**Figure 3:**
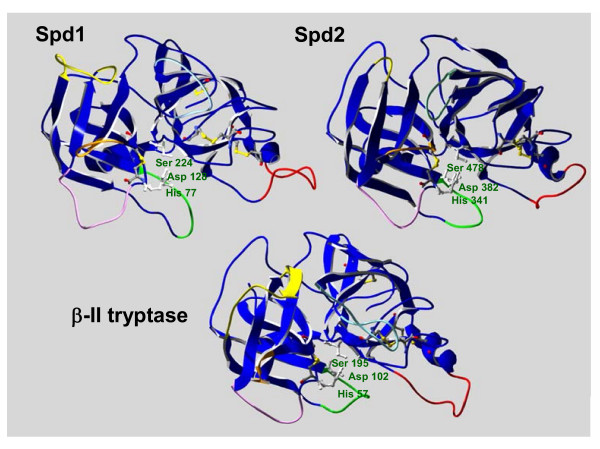
Homology models of the catalytic domains of polyserase-3 and β-II tryptase. The structural modeling of Spd-1 and Spd-2 reveals the high degree of similarity with the serine protease β-II tryptase. The twelve loops (six in each serine protease module) potentially involved in the dimerization of polyserase-3 as well as the 6 loops involved in β-II tryptase tetramerization are represented using the color code previously described by Sommerhoff *et al. 1999 *(33). The molecules are oriented towards the active site and the three residues that compose the catalytic triad of each serine protease are indicated. The backbone and side chains of the disulfide bonds are represented in CPK color scheme.

The amino acid sequence similarity between each serine protease domain of polyserase-3 and serine proteases whose three-dimensional structures are available, opened the possibility of performing their structural modeling (Fig. 3). This analysis revealed a significant degree of similarity between both domains of polyserase-3 and some members of the tryptase family, such as human β-tryptase II [33]. Thus, in the predicted structure there is a loop that surrounds a calcium ion in most serine proteases (shown in yellow in Fig. 3) [34], although in the case of Spd1 and β-II tryptase is shorter, and it does not exist in Spd2, suggesting that polyserase-3 does not requires calcium for its activity. Apart from the disulfide bonds deduced from the alignment of polyserase-3 sequence with other serine proteases, the structural model of polyserase-3 predicts the existence of seven additional disulfide bonds. Four of these bonds are predicted to occur within Spd1, and the eight cysteine residues involved would be Cys62-Cys78, Cys158-Cys230, Cys187-Cys209, and Cys220-Cys249. Equivalent disulfide bonds are predicted in the structure of the human β-tryptase II (Fig. 3). The three remaining bonds would occur within Spd2, and the involved residues would be Cys326-Cys342, Cys444-Cys464, and Cys474-Cys502 (Fig. 3).

### Polyserase-3 is a secreted and non-glycosylated protein

**Figure 4:**
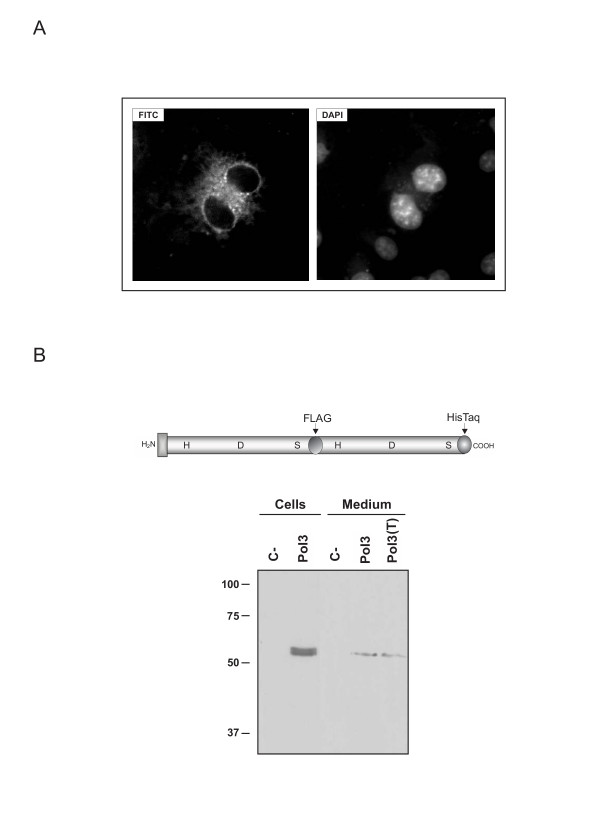
Immunocytochemical detection and analysis of recombinant polyserase-3 expression in human cell lines. *A*, image captures by fluorescence microscopy of 293-EBNA cells transfected with pCEP-pol3 vector and incubated with an anti-FLAG antibody, followed by incubation with a secondary fluorescein-conjugated antibody (FITC). This signal was not detected in cells transfected with an empty vector. The observed cytoplasmic fluorescence indicates that polyserase-3 is not a membrane-anchored protease. DNA in the cell nucleus was visualized with DAPI. *B*, representation of the recombinant polyserase-3 containing the indicated epitopes, and Western blot analysis of 293-EBNA cells and conditioned medium using an anti-FLAG antibody. Equivalent results were obtained using an anti His-Tag antibody (not shown). C- indicates cells or conditioned medium from cells transfected with the empty vector, and T indicates sample treated with 1 μg/mL tunicamycin. The concentration of the SDS-PAGE gel was 12%, and the molecular mass markers in kDa are indicated on the left.

The pCEP-pol3 vector was used to transfect 293-EBNA cells. Immuno-localization experiments using an anti-FLAG antibody showed a strong eccentric perinuclear signal (Fig. 4A). Moreover, and consistent with the absence of a membrane localization motif in the polyserase-3 sequence, we did not find any evidence of immunostaining at the cell surface. Similar results were obtained using HeLa cells transfected with the pCEP-pol3 vector (not shown). Likewise, the positive signal was only detected if cells were previously permeabilized using Triton X-100. This situation resembles that observed for polyserase-2 [3] and differs from that of polyserase-1, which is a membrane-bound polyprotease [9]. All these findings strongly suggest that polyserase-3 is a secreted polyserine protease. This possibility was further confirmed by Western blot analysis of the conditioned medium prepared from pCEP-pol3 transfected cells (Fig. 4B). In fact, the anti-FLAG antibody detected one immunoreactive band of about 55 kDa, which fits with the expected size for unprocessed polyserase-3. On the other hand, a doublet of similar size, which likely represents the protein with or without signal peptide, was detected in cell fractions, but none of them were present in cells transfected with the empty vector. To evaluate the possibility that the FLAG epitope could hamper the proper processing of the two serine protease domains of polyserase-3, we generated a construct lacking this epitope but keeping a HisTag tail at the C-terminus. Western blot analysis using an anti-HisTag antibody showed the same result as above (not shown), thereby confirming that both protease domains of polyserase-3 remain as integral parts of the same polypeptide chain. Additionally, and contrary to polyserase-2, the mobility of the band detected with this anti-HisTag antibody was not altered in the presence of tunicamycin, an inhibitor of N-glycosylation, suggesting that polyserase-3 is a non-glycosylated protein. Consistent with this, analysis of the polyserase-3 sequence using the NetNGlyc 1.0 Server [35] predicted that the only putative N-glycosylation site present in this protein (Asn543), would not be effectively glycosylated.

### Production, purification and enzymatic assays of full-length polyserase-3 and its serine-protease domains

**Figure 5:**
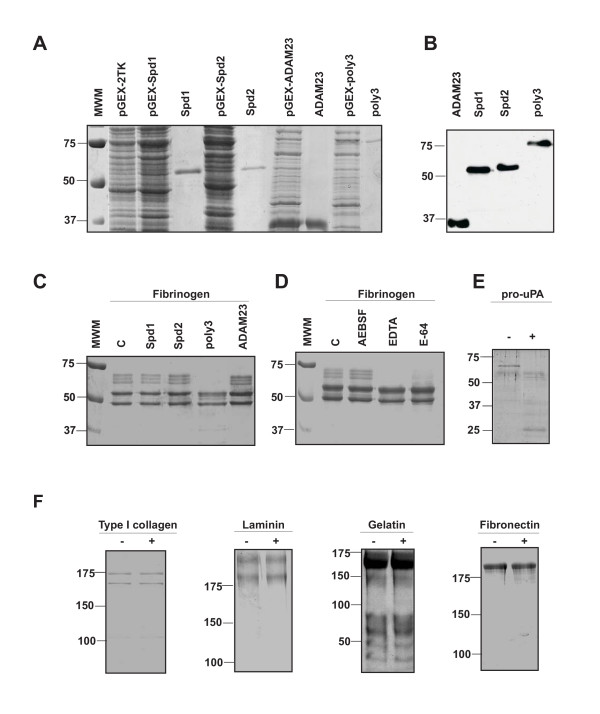
Production and activity assays of recombinant Spd-1, Spd-2, and polyserase-3. *A*, 5 μL of bacterial extract transformed with the plasmids pGEX-Spd1 (lane3), pGEX-Spd2 (lane 5) and pGEX-pol3 (lane 9), and the corresponding purified fusion proteins (Spd1, lane 4; Spd2, lane 6, and polyserase3, lane 10). Lane 2, bacterial extract transformed with an empty pGEX-2TK. Lanes 7 and 8 correspond to induction and purification of ADAM23 disintegrin domain, respectively. Lane 1, molecular size markers, whose sizes in kDa are indicated on the left. *B*, Western blot analysis of the purified fusion proteins using an anti GST antibody. *C*, degradation of fibrinogen by the indicated fusion proteins. C, control, corresponds to incubation of fibrinogen without any recombinant protein. *D*, Inhibition analysis of polyserase-3 after preincubation with AEBSF (0.1 mM), EDTA (2 mM) and E-64 (10 μM). *E*, digestion of pro-uPA by purified polyserase-3. C indicates incubation of pro-uPA alone. *F*, incubation of different extracellular proteins with purified polyserase-3.

To produce the recombinant proteins, we first transformed *E. coli *strain BL21(DE3) pLysE with plasmids pGEX-pol3Spd1, pGEX-pol3Spd2, and pGEX-pol3. Moreover, we used ADAM23 disintegrin domain fused to GST to verify the purification processes as well as a negative control in the enzymatic assays [36]. After IPTG induction of bacterial cells transformed with these plasmids, fusion proteins of the expected size (55, 57, 83 and 36 kDa respectively) were detected by SDS-PAGE (Fig. 5A). Once the purification process was carried out as indicated above, the fusion proteins were visualized by SDS-PAGE (Fig. 5A), and their identities confirmed by Western blotting using an anti-GST antibody (Fig. 5B). We next incubated the recombinant protein with a variety of different endogenous proteins including type I collagen, type I laminin, gelatin, pro-uPA and fibrinogen were treated with the recombinant proteases. Among all these potential extracellular substrates, fibrinogen and pro-UPA were clearly degraded by the entire polyserase-3, but not by its serine protease domains produced as independent proteins (Fig. 5C–E, and data not shown). This activity was abolished by preincubating the enzyme with AEBSF, a serine protease inhibitor, but not when the enzyme was treated with inhibitors of other classes of proteases (Fig. 5D). These data provide additional support to the proposal of this enzyme as a catalytically active serine protease. Moreover, SDS-PAGE analysis of the recombinant polyserase-3 incubated for 16 h at 37°C also indicates that the enzyme is released from the GST-moiety (data not shown), which could be due to an autoactivation process of polyserase-3, similarly to fusion proteins containing the catalytic domain of matriptase-1 [37] or matriptase-2 [28].

### Polyserase-3 may form active dimers

**Figure 6:**
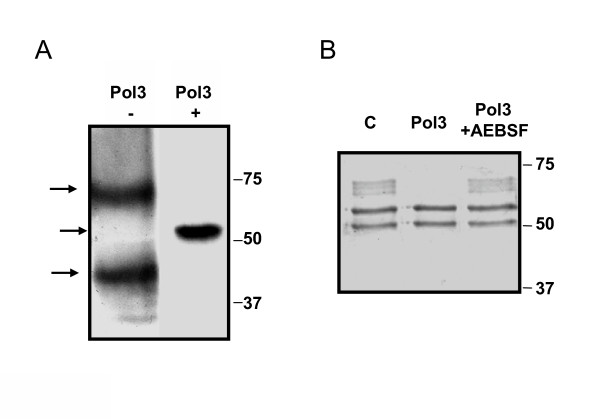
Polyserase-3 may form active dimers. *A*, Western blot analysis of His-tagged polyserase-3 in the absence (-) or presence (+) of 2% 2-mercaptoethanol, using an anti-HisTag antibody. Molecular size markers are indicated on the right, and detected bands are indicated with arrows on the left. *B*, degradation of fibrinogen by His-tagged polyserase 3 in the absence or presence of the serine protease inhibitor AEBSF.

Some tryptases, which share several features with polyserase-3, can form active tetramers. Moreover, other members of this group of serine proteases, such as mouse mast cell tryptase [38], are able to degrade the α-chain of fibrinogen when forming tetramers in a similar manner to that shown herein for polyserase-3. On this basis, we hypothesized that two polyserase-3 molecules could associate to produce a protein structurally equivalent to the tetramers formed by this type of tryptases. To evaluate this question, we produced a recombinant protein containing a 6xHisTag tail at the N-terminus. This new recombinant polyserase-3, purified as described in Experimental Procedures, was incubated in the presence or absence of a reducing agent (2-mercaptoethanol) and detected by Western blot using an anti-HisTag antibody (Fig. 6A). The presence of two immunoreactive bands in native conditions and one band of the expected size in the sample containing the denaturing reagent suggested that polyserase-3 forms dimers which seems to be stabilized by disulfide bridges, as reported for the dog mast cell protease-3 [39]. Interestingly, this recombinant 6xHis tagged polyserase-3 degrades fibrinogen similarly to the GST-polyserase-3 protein (Fig. 6B), suggesting that fibrinogen degradation by the fusion protein, could occur once polyserase-3 is released from the GST.

### Analysis of polyserase-3 expression in human tissues

**Figure 7:**
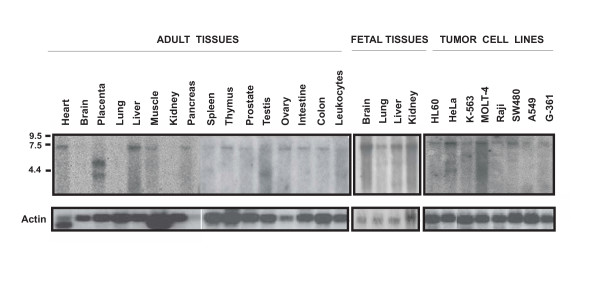
Analysis of polyserase-3 expression in human tissues and tumor cell lines. Approximately 2 μg of polyadenylated RNA from the indicated tissues or tumor cell lines was hybridized with a probe specific for polyserase-3. The position of the RNA markers is shown. The filters were subsequently hybridized with an actin probe to ascertain the differences in RNA loading among the different tissues.

A cDNA probe specific for human polyserase-3 was used to hybridize Northern blots containing poly(A)+ RNAs from a variety of human fetal and adult tissues, and tumor cell lines (Fig. 7). This analysis showed a band of about 7.5 kb in different adult tissues including liver, heart, testis, ovary, intestine, colon and leukocytes. A band of the same size was observed in all analyzed fetal tissues such as kidney, liver, lung and brain. This transcript was also detected in human cancer cell lines, including HeLa (cervix adenocarcinoma), MOLT-4 (lymphoblastic leukaemia), and SW480 (colon adenocarcinoma). Bioinformatic analysis using different programs available at the NIX tool [40], predicts a transcript of around 8 kb for this gene, suggesting that the higher band observed in Fig. 7, likely corresponds to a full-length polyserase-3 transcript. However, other transcripts of 5.2 kb and 4.2 kb were observed at placenta, testis, HeLa and MOLT-4 cells. The presence of these transcripts of smaller size suggests that the polyserase-3 gene could also be regulated through alternative splicing events which may produce a protein without one of its serine protease domains. This mRNA processing of a multidomain protease has also been described for polyserases -1 and -2 [3,9].

## Discussion

In this work, we have performed an exhaustive bioinformatic analysis of the human genome to try to identify new serine proteases that could contain different catalytic domains within the same polypeptide chain. These bioinformatic searches led us to find a region in chromosome 16p11.2 putatively encoding a new polyprotease. After completing the cloning process using liver cDNA as template, we confirmed that the identified sequence was a new polyserine proteinase that we called polyserase-3 to underline its structural relationship with the previously described polyserases-1 and -2 [3,9]. However, the polyserase-3 architecture is less complex than the exhibited by the two other human polyproteases. Thus, this new polyserase is composed of two serine protease domains preceded by a signal peptide, whereas both polyserase-1 and polyserase-2 contain three catalytic domains in a single polypeptide chain.

A comparative structural analysis also revealed that polyserase-3 is more closely related to polyserase-2 than to polyserase-1. Thus, and similar to polyserase-2, polyserase-3 is a secreted soluble protein that lacks additional domains found in polyserase-1 such as a type II transmembrane sequence and a low-density lipoprotein receptor motif. Likewise, the serine protease domains of polyserase-2 and polyserase-3 remain as integral parts of the same molecule, whereas polyserase-1 undergoes a series of post-translational processing events that release the three protease domains from the initial translation product [9]. The structural basis for these differences may derive from the fact that both protease domains of polyserase-3, as well as the second and third protease domains of polyserase-2, are preceded by a region lacking the consensus activation motif Arg-Ile-Val-Gly-Gly, characteristic of serine proteases. Therefore, it is unlikely that these domains can be separated from the original polypeptide chain by a trypsin-like protease. Nevertheless, the possibility that polyserase-3 can be activated under specific circumstances through alternative mechanisms such as those operating in α-tryptase [41,42], can not be ruled out.

The phylogenetic analysis of human serine proteases whose sequence is available, also revealed the relationship between polyserases-2 and -3, since the different serine protease domains of both polyproteases are grouped together and form a branch equally distant from members of the TTSP and the tryptase/prostasin families. Interestingly, analysis of the gene structure and organization of polyserases-2 and -3 showed common features with these two groups of serine proteases. Therefore, it is possible that ancestors of both TTSPs and tryptase/prostasin contributed to the formation of these polyserases through recombination or exon swapping events. Likewise, the gene encoding polyserase-3 maps very close to the polyserase-2 and prostasin genes at chromosome 16p11.2, a region linked to genetic abnormalities whose loci remain unidentified. These pathologies include paroxysmal kinesigenic choreoathetosis [43] and autosomal dominant myxomatous mitral valve prolapse [44], opening the possibility that the identified human polyserases could be implicated in the development of these diseases. Nevertheless, beyond all these similarities between human polyserases-2 and -3, clear differences were also detected between them. Thus, we have previously reported that polyserase-2 is a glycosylated protein that only shows catalytic activity in its first serine protease domain. By contrast, polyserase-3 is a secreted and non-glycosylated enzyme.

As an initial step towards the functional characterization of polyserase-3, we analyzed its expression profile in different human tissues. These studies revealed additional differences between polyserase-3 and the previously described human polyserases in their patterns of expression in different tissues and cancer cell lines. Thus, polyserase-3 is mainly expressed in adult heart, liver, intestine, ovary and testis, as well as in all analyzed fetal tissues including kidney, brain, liver and lung. By contrast, polyserase-2 is predominantly detected in adult skeletal muscle, liver, placenta, prostate and heart, as well as in fetal kidney but not in fetal lung, liver or brain. It is also noteworthy that several human tumor cell lines also express this new polyprotease, a feature shared with the previously described human polyserases, opening the possibility that these complex serine proteases could mediate proteolytic processes associated with tumor development or progression [26,45].

To evaluate the possibility that polyserase-3 is an active enzyme with ability to perform these proteolytic events, we undertook the production of the entire protein as well as its two serine protease domains as independent proteins. The activity assays showed that the complete polyserase-3 is able to degrade some substrates present in the extracellular matrix such as fibrinogen and pro-uPA. These results suggest that this enzyme could contribute to tumor progression either through the degradation of extracellular matrix proteins or the activation of other components including different tumor-associated proteases [46]. Contrary to the situation with the entire protein, the two serine protease domains produced as independent proteins did not show any apparent proteolytic activity against the substrates indicated above. Interestingly, members of the tryptase family – that show significant degree of structural similarity with both protease domains of polyserase-3- are secreted as monomers and must form tetramers to carry out the catalysis [33]. These facts prompted us to evaluate whether this enzyme could dimerize to generate a protein with four potential active sites, and whose quaternary structure could be similar to that formed by the tryptases. To analyze this possibility, we compared the electrophoretic mobility of the recombinant protein under non-reducing and reducing conditions. This assay would indicate that approximately half of the purified polyserase-3 may form active dimers which are likely stabilized through the formation of disulfide bonds. The information derived from the three-dimensional models generated for Spd1 and Spd2 was also consistent with this possibility. Thus, these models, together with predictions based on amino acid sequence alignments, suggest that polyserase-3 possesses a total of nine intrachain disulfide bonds. However, there are three free cysteines (Cys189, 293 and 543) that could be involved in the stabilization of the polyserase-3 dimer through the formation of disulfide bonds. The stability of the dimer would be further maintained by a series of conserved tryptophan residues (Trp 48, 50, 159 and 237 in Spd-1, and Trp 312, 314, 417 and 490 in Spd2) that have been reported to be necessary for mouse mast cell tryptase dimerization and activity [38]. Interestingly, this protease can also cleave the α-chain of fibrinogen as demonstrated herein for polyserase-3. Due to the homology of polyserase-3 and β-II tryptase, we cannot rule out the existence of further interactions involving hydrogen bonds and salt bridges and participating in the dimer formation through the six loops (code-colored in Fig. 5A) previously described for β-II tryptase [33]. Regarding the fact that fibrinogen cannot be cleaved by Spd1 or Spd2 produced as independent proteins, we can speculate that the presence of three aspartic acid residues (Asp164, 166 and 169) in a loop of Spd1 could form a negatively charged anchoring site that would compete with the substrate binding pocket when the protein is in a monomer state. Stabilization of this loop in the dimer state, probably by Spd2, would grant access for the substrate to the active site. These acidic residues seem also to be important for β-II tryptase, whose enzymatic activity is totally abolished in the monomer state [33]. Nevertheless, further functional studies will be necessary to verify these predictions in the case of polyserase-3.

## Conclusion

The identification of the third human polyserase allows establishing the polyserases as a group of enzymes containing different tandem serine protease domains. These findings raise new questions about the functions of these intriguing polyenzymes and their possible involvement in human diseases. In particular, the search for the *in vivo *substrates of polyserase-3 and the generation of mice deficient in this gene would contribute to ascertain the relevance of this enzyme in both normal and pathological conditions. Finally, the resolution of the three- dimensional structure of polyserase-3 and that of the remaining human polyserases, could help to understand the functional relevance of the presence of several catalytic domains within the same polypeptide chain.

## Methods

### Materials

Nylon filters containing polyadenylated RNAs from human tissues and tumor cell lines were purchased from Clontech. Restriction endonucleases and different reagents for molecular cloning, including the Expand™ High Fidelity PCR system and the Thermoscript reverse transcription-PCR system were from Roche Applied Science. DNA probes were radiolabeled using [α-^32^P]dCTP (3000 Ci/mmol) and a random-priming kit from Amersham Biosciences.

### Bioinformatic analysis and cDNA cloning

Human polyserase-2 cDNA and the BLAST program were used to query regions in the human genome that could be predicted as new polyserase genes. These searches allowed us to identify a putative region in chromosome 16p11.2 that encoded a new enzyme with two seine protease domains. Then, a PCR-based strategy was used to clone the full-length cDNA for this novel polyprotease. To this end, specific oligonucleotides derived from the genomic sequences were used to screen a panel of human cDNA libraries for transcripts corresponding to this new polyserine protease. The sequences of the designed primers were pol3-f1 (forward) 5'- GGCTGCCCTGCAGTTGCC-3', and pol3-r1 (reverse) 5'-CAGGTGGTGGTCAGTAGGG-3' for polyserase-3. All PCRs were performed in a GeneAmp 2400 PCR system (PerkinElmer Life Sciences) for 40 cycles of denaturation (94°C, 20 s), annealing (63°C, 20 s), and extension (68°C, 60 s). After cloning of the PCR-amplified products in pBlueScriptII (Invitrogen), their identities were confirmed by nucleotide sequencing using the kit DR terminator *Taq*FS and the automatic DNA sequencer ABI-PRISM 310 (Perkin-Elmer Life Sciences). Sequence analysis of the RACE-extended cDNA clones led us to complete the identification of the new polyserase. Finally, the full-length cDNA was obtained by PCR using the primers ATGpol3 (forward) 5'-ATGAAGTGGTG CTGGGGCCCA-3', ENDpol3 (reverse), 5'-TCAGCAGCTGGTTGGTTGGCT-3'. PCR conditions were as above, but with 280 s of extension. Nucleotide and protein sequence analysis were carried out using different programs available [35,40].

### Northern-blot analysis

Nylon filters containing poly(A)^+ ^RNAs of diverse human tissues were prehybridized at 42°C for 3 h in 50% formamide, 5× SSPE, 10× Denhardt's solution, 2% SDS, and 100 μg/ml of denatured herring sperm DNA. Hybridization was performed with a radiolabeled 628 pb *Eco*RI-*Bam*HI fragment of polyserase-3. After hybridization for 20 h under the same conditions, filters were washed with 0.1× SSC, 0.1% SDS for 2 h at 50°C, and exposed to autoradiography.

### Construction of expression vectors and purification of recombinant proteins

To analyze the expression of polyserase-3 in eukaryotic cells, the full-length cDNA encoding this protein was PCR-amplified and cloned between the *Hind*III and *Not*I sites of a modified pCEP4 expression vector (Invitrogen), which facilitated to add a HisTag tail at the C-terminus of the recombinant protein. The oligonucleotides used to do this were 5'-TTA**AAGCTT**ATGAAGTGGTGCTGGGGCC-3' (forward) and 5'-TGT**GCGGCCGC**GCAGCTGGTTGGTTGGCTA-3' (reverse) where the *Hind*III and *Not*I sites are indicated in bold. A *Bss*HII site was created in the coding sequence (positions 809 to 814) which allowed us to introduce a FLAG epitope between the two serine protease domains, using the oligonucleotides 5'-CGCGCGACTACAAGGACGACGATGACAAG-3' and 5'-CGCGCTTGTCATCGTCGTCCTTGTAGTCTG-3'. The resulting vector, pCEP-pol3, was transfected into HeLa and 293-EBNA cells using the LipofectAMINE reagent (Life Technologies, Inc.). When indicated, tunicamycin was added to the cells at a final concentration of 1 μg/mL. Expression of each independent serine protease domain as well as the entire protease in bacterial cells was carried out using the pGEX-2TK vector (Amersham Biosciences). To this end, the first serine protease domain was PCR-amplified from pCEP-pol3 using the oligonucleotides 5'-GAGGGCAACACAGTCCCTGGCGAG-3' (forward) and 5'-GTAGAGGCCCCAGAGACCCGA-3' (reverse), and cloned into the *Sma*I site of the pGEX vector to generate pGEX-pol3Spd1. Similarly, the second serine protease domain was amplified by PCR using the oligonucleotides 5'-TCGGGTCTCTGGGGCCTCTAC-3' (forward) and 5'-GTGATGGTGATGGTGATGTGC-3' (reverse) and cloned as above to prepare the construct pGEX-pol3Spd2. To produce the entire protease, a PCR-amplification was carried out with the oligonucleotides 5'-GAGGGCAACACAGTCCCTGGCGAG-3' and 5'-GTGATGGTGATGGTGATGTGC-3' and the PCR product was cloned as above to get pGEX-pol3 vector. A pGEX plasmid expressing ADAM23 disintegrin domain was used as control to assess quality in the further purification process of the fusion proteins, and as negative control of the serine protease activity in the enzymatic assays [36]. Plasmids were transformed into BL21(DE3) pLysE *Escherichia coli *cells and expression was induced at 19°C using 0.4 mM of isopropyl-β-D-thiogalactoside (IPTG). After that, cells were collected, lysed and centrifuged, and the soluble fractions containing the recombinant proteins were purified as follows. A glutathione-Sepharose 4B (Amersham Biosciences) was initially used and the eluted proteins were subsequently loaded in a gel filtration column (Superdex 200, Amersham Biosciences). The quality of the purification process was followed by SDS-PAGE and Western blot analyses, using an anti-GST antibody (Amersham Biosciences). To evaluate the possibility that polyserase-3 forms dimers, the full-length cDNA was amplified using the oligonucleotides 5'-GCA**AGATCT**AACACAGTCCCTGGCGA GTGG-3' (forward) and 5'-TGC**AAGCTT**TCAGCAGCTGGTTGGTTGGCTTAT-3' (reverse), where the *Bgl*II and *Hind*III sites are indicated in bold. The PCR-product was digested with these restriction enzymes and cloned between these positions in pRSETB, which add a 6xHisTag tail at the N-terminus of the protein. The resulting vector (pRSETB-pol3) was transformed into BL21(DE3) pLysE. The production of the recombinant protein was as above; whereas the purification was carried out using a Ni-NTA column (Qiagen), and an anti 6xHisTag antibody (Qiagen) was used to detect the produced protein.

### Activity assays

The catalytic activity of the recombinant proteins was analyzed using a panel of different proteins as potential substrates including type I collagen, type I gelatin, type I laminin, pro-uPA and fibrinogen. The assays were carried out with 5 μg of each protein in a buffer containing 50 mM Tris-HCl pH 7.4 and 150 mM NaCl, during 16 h at 37°C. The enzyme/substrate ratio (w/w) used in these experiments was 1/100. The resulting material was subjected to SDS-PAGE analysis. For inhibition assays, polyserase-3 was preincubated with AEBSF (0.1 mM), EDTA (2 mM), and E-64 (10 μM) for 30 min at 37°C, and then incubations were performed at the same conditions as above.

### Homology modeling

A three-dimensional model of each polyserase-3 serine protease domains was calculated using Swiss-Model, a semiautomated modeling server [47], and analyzed with the Swiss-Pdb Viewer. The amino acid sequence of each serine protease domain was compared with the sequences of the protein structures deposited in the Protein Data Bank. After analyzing structures of non-redundant proteins that had the highest structural quality and significant sequence similarity with each polyserase-3 catalytic domain, we chose the human β-tryptase (1a01), matriptase-1 (1eaxa) and human plasmin (1bmla) as templates. The templates were superimposed and aligned structurally. The quality of the resulting models was verified manually with Swiss-Pdb Viewer. The figures were rendered with POV-Ray [48].

### Immunocytochemical analysis

After transfection, HeLa and 293-EBNA cells were fixed with 4% paraformaldehyde in PBS. Then, cells were permeabilized for 5 min with 0.2% Triton X-100 in PBS. Blocking was carried out with 15% fetal bovine serum in the same buffer. Blocked slices were incubated for 2 h with different dilutions of the primary anti-FLAG antibody, followed by 1 h incubation with a secondary fluorescein-conjugated goat anti-mouse antibody. Slides were coverslipped in the presence of Vectashield medium (Vector Laboratories) containing 4'-6'-diamidino-2-phenylindole hydrochloride (DAPI) and imaged by fluorescence microscopy.

## Abbreviations

The abbreviations used are: AEBSF, 4-(2-aminoethyl)-benzenesulfonyl fluoride; bp, base pair(s); DAPI, 4'-6'-diamidino-2-phenylindole hydrochloride; GST, glutathione S-transferase; PAGE, polyacrylamide gel electrophoresis; PCR, polymerase chain reaction; RACE, rapid amplification of cDNA ends; RT, reverse transcription; SDS, sodium dodecyl sulfate; Spd, serine protease domain; TTSP, type II transmembrane serine proteinase; uPA, urokinase-type plasminogen activator.

## Authors' contributions

S.C. participated in the design of the study and performed the identification and cloning of polyserase-3. He also contributed to the elaboration of the manuscript. J.-R.P. carried out the expression of the recombinant protein in different systems and performed the molecular modelling studies. M.L. collaborated in the molecular cloning studies and performed tissue expression analysis of polyserase-3. V.Q. participated in the purification and enzymatic analysis of the recombinant proteins. A. M.-P. carried out the evolutionary analysis of polyserase-3. C.G. performed studies with the different cell lines. C.L.-O. conceived the study, participated in its design and wrote most sections of the manuscript. All authors read and approved the final manuscript.
